# Exploring MOF-Derived CuO/rGO Heterostructures for Highly Efficient Room Temperature CO_2_ Sensors

**DOI:** 10.1021/acssensors.4c01397

**Published:** 2024-09-18

**Authors:** Toton Haldar, Jia-Wei Shiu, Ren-Xuan Yang, Wei-Qi Wang, Hsin-Ting Wu, Hsu-I Mao, Chin-Wen Chen, Chi-Hua Yu

**Affiliations:** † Department of Engineering Science, National Cheng Kung University, Tainan 701401, Taiwan; ‡ Department of Molecular Science and Engineering, National Taipei University of Technology, Taipei 106344, Taiwan; § Institute of Environmental Engineering and Management, National Taipei University of Technology, Taipei 106344, Taiwan

**Keywords:** metal organic framework, p-p heterojunction, graphene oxide, DFT, HOMO−LUMO energy gap

## Abstract

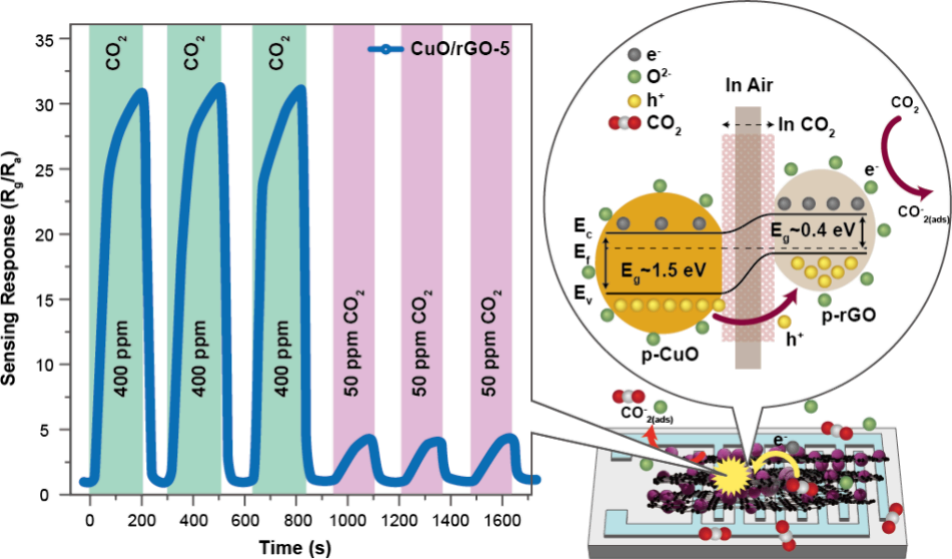

In response to the urgent need for advanced climate change mitigation tools, this study introduces an innovative CO_2_ gas sensor based on p-p-type heterostructures designed for effective operation at room temperature. This sensor represents a significant step forward, utilizing the synergistic effects of p-p heterojunctions to enhance the effective interfacial area, thereby improving sensitivity. The incorporation of CuO nanoparticles and rGO sheets also optimizes gas transport channels, enhancing the sensor’s performance. Our CuO/rGO heterostructures, with 5 wt % rGO, have shown a notable maximum response of 39.6–500 ppm of CO_2_ at 25 °C, and a low detection limit of 2 ppm, indicating their potential as high-performance, room-temperature CO_2_ sensors. The prepared sensor demonstrates long-term stability, maintaining 98% of its initial performance over a 30-day period when tested at 1-day intervals. Additionally, the sensor remains stable under conditions of over 40% relative humidity. Furthermore, a first-principles study provides insights into the interaction mechanisms with CO_2_ molecules, enhancing our understanding of the sensor’s operation. This research contributes to the development of CO_2_ monitoring solutions, offering a practical and cost-effective approach to environmental monitoring in the context of global climate change efforts.

Achieving carbon neutrality requires balancing emitted carbon with removal or offsetting, targeting net zero emissions to combat climate change.^1,2^ Human activities like fossil fuel combustion and deforestation have significantly increased CO_2_ levels, intensifying global warming.^3−5^ Effective CO_2_ detection is vital, enabling precise monitoring and management of greenhouse gases and informing reduction strategies. This necessitates the development of sensitive and economical gas sensors to address environmental and health risks.^6−9^

However, CO_2_ significantly impacts indoor air quality by increasing concentrations of hazardous pollutants such as volatile organic compounds (VOCs), which can worsen health risks.^10,11^ Elevated CO_2_ levels enhance the greenhouse effect and deteriorate indoor environments, as shown in a 2021 study by Peng and Jimenez.^12^ This study links high CO_2_ levels to poor ventilation and potential pathogen transmission, including SARS-CoV-2.^12^ In contrast, administering CO_2_ under 300 ppm can promote plant growth by reducing microbial activity, while food storage areas may have CO_2_ levels up to 25% to preserve freshness by inhibiting microbes.^13−15^ This highlights the critical need for advanced room temperature CO_2_ sensors for public health and specific applications such as agriculture and food preservation.

To enhance room-temperature gas sensing, a promising approach involves integrating metal oxides with higher electric charge carriers into reduced graphene oxide (rGO).^16,17^ This technique prevents the aggregation of rGO sheets by enhancing electrostatically repulsive forces. Metal oxides such as ZnO, TiO_2_, SnO_2_, and WO_3_, when combined with rGO and other 2D materials, form heterojunction materials that significantly improve the sensitivity of gas sensors.^18−22^ For example, Kim et al.^23^ developed a ZnO-graphene heterostructure CO_2_ sensor, achieving a 78% response at 400 °C and a 1000 ppm detection limit. Amiri et al.,^24^ enhanced CO_2_ detection using graphene-TiO_2_ nanocomposite layers (G-TiO_2_–NCLs), achieving significant response improvements at 200 °C. Lee et al.^25^ introduced a SnO_2_-rGO nanocomposite sensor that demonstrated a strong linear response to CO_2_ with a 5 ppm detection limit, offering low-cost and energy-efficient advantages. Tripathy et al.,^26^ developed PEI/NrGO/ZNR sensors, showing a response increase from 5.6 to 16.98% with rising CO_2_ levels, indicating potential for monitoring conditions like chronic obstructive pulmonary disease (COPD).

Copper oxide (CuO) is a p-type semiconductor with a band gap ranging from 1.2 to 1.9 eV, making it a versatile material suitable for various applications including catalysis, field emission devices, electrochemical cells, and gas sensors.^27,28^ Meanwhile, metal–organic frameworks (MOFs), known for their adjustable topology and porosity through the selection of metal nodes and organic linkers, serve as excellent templates for creating various porous structures such as nanospheres, hierarchical flowers, and nanorods.^29−32^ However, pure CuO gas sensors suffer from low sensitivity, a limitation often addressed by using MOFs as sacrificial templates to produce metal oxides and rGO with enhanced porosity and specific morphologies under controlled conditions.^33,34^ Recently, this approach has become increasingly popular due to the advantageous properties of MOF-derived metal oxide, which enhance performance in sensor applications.^35,36^ For example, Chen et al.,^37^ utilized the MOF template to develop tourmaline@ZnO nanostructures, significantly enhancing n-butanol gas sensing by improving the surface area and electron transport efficiency. Similarly, Sun et al.,^38^ demonstrate that Ce-doped In_2_O_3_ hollow nanoboxes derived from MOFs significantly enhance gas sensing, exhibiting rapid response and high sensitivity to formaldehyde.

In response to the critical need for advanced gas sensing technologies, particularly for the detection of CO_2_ at room temperature, this study focuses on the development and evaluation of MOF-derived CuO/rGO heterostructures. Leveraging the novel combination of CuO, a metal oxide renowned for its gas sensing capabilities, with rGO, known for its exceptional properties and p-type semiconductor behavior, we aim to synthesize p-type heterostructures by using a simple solvothermal method. The objective is to harness the synergistic potential of CuO and rGO to enhance the gas sensing performance. Comprehensive characterization of these MOF-derived heterostructures will ascertain their physical and chemical attributes, crucial for their function as sensitive and selective CO_2_ gas sensors. The study methodically evaluates the performance of these heterostructures as sensing materials, focusing on their sensitivity, selectivity, and response times to various gases, including CO_2_, NH_3_, CO, NO_2_, H_2_S, CH_4_, and C_2_H_5_OH, across concentrations from 50 to 500 ppm. This encompasses a range of indoor and outdoor environmental scenarios. Additionally, density functional theory (DFT) analysis will be utilized to analyze the molecular interactions between the heterostructures and CO_2_, enhancing our understanding of the detection mechanisms.

## Results and Discussion

### Structure and Compositional Analysis

#### X-ray Diffraction

**Figure 1:**
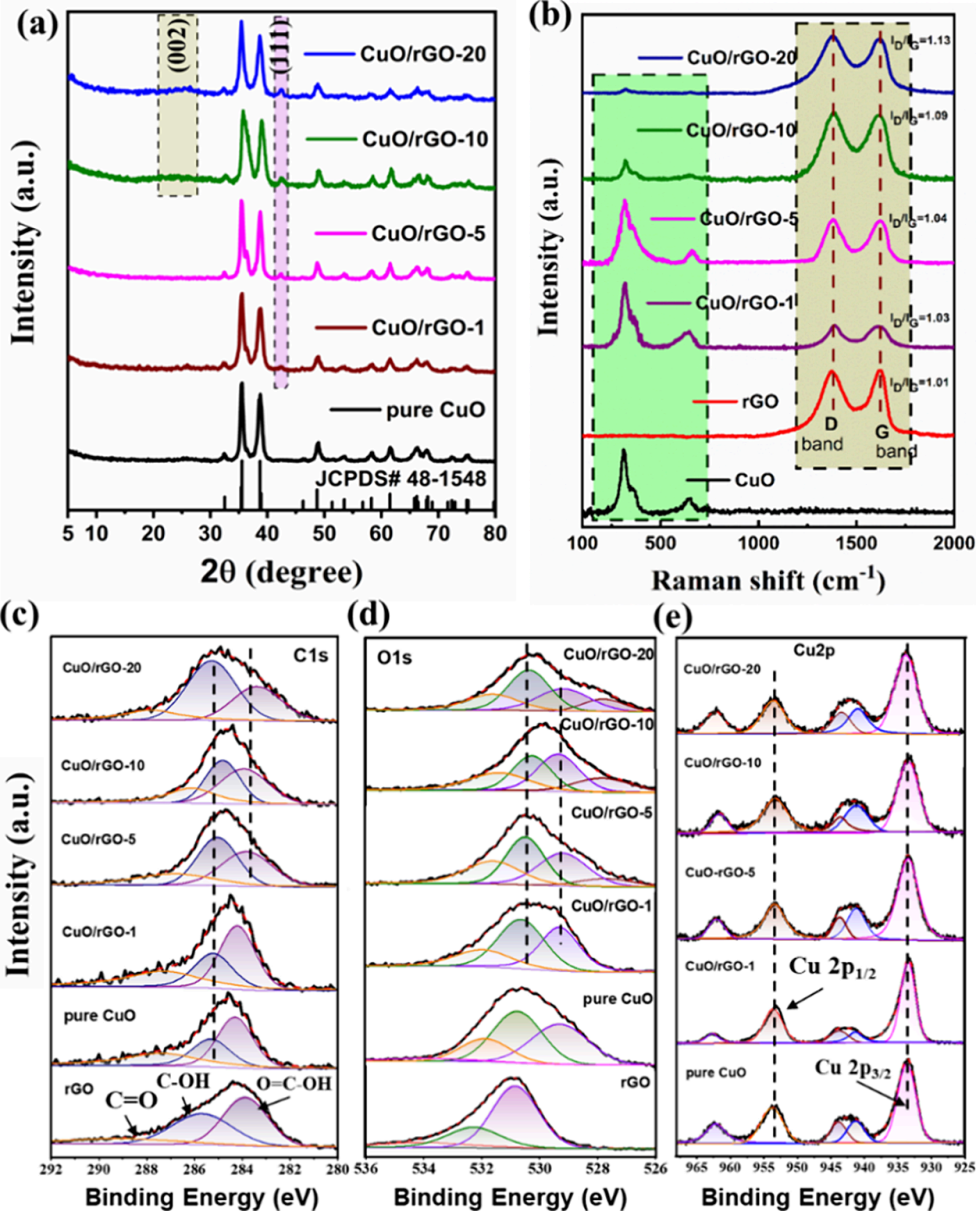
(a) XRD spectra of pure CuO, CuO/rGO-1, CuO/rGO-5, CuO/rGO-10, and CuO/rGO-20 heterostructures and (b) Raman spectra of pure CuO, GO, CuO/rGO-1, CuO/rGO-5, CuO/rGO-10, and CuO/rGO-20 heterostructures, XPS spectra of rGO, pure CuO, CuO/rGO-1, 5, 10, and 20 heterostructure composites (c) C 1s, (d) O 1s, and (e) Cu 2p.

Solvothermally prepared CuO-MOF and CuO/rGO MOF powders were transformed into CuO/rGO heterostructures using a simple thermal decomposition methodology. The detailed experimental process is presented in the Supporting Information, as depicted in Figure S1. The crystalline structure of the synthesized pure CuO and CuO/rGO heterostructures, incorporating concentrations of 1, 5, 10, and 20 wt %, were recorded through powder X-ray diffraction (PXRD) analysis (Figure 1a). The PXRD patterns of pristine CuO resonated with the monoclinic-phase characteristics (JCPDS No. 45-1548), revealing peaks at 2θ values of 35.5° and 38.7°, which are emblematic of the (111̅) and (111) planes, respectively, thereby highlighting the crystalline integrity of CuO. Further, peaks positioned at 2θ angles of 32.4°, 46.3°, 48.6°, 51.2°, 53.6°, 58.9°, 61.6°, 66.3°, 68.2°, and 72.5° were ascribed to the monoclinic phase’s (110), (112̅), (202̅), (112), (020), (202), (311̅), (113̅), (220), and (311) planes, respectively. The CuO/rGO heterostructures for the higher content of rGO (10 wt % of rGO and more) demonstrated a pronounced peak at 25°, attributable to the rGO peak. In contrast, for the lower content of rGO in CuO/rGO heterostructure (i.e., 1 and 5 wt % rGO), there were no detectable peaks for rGO. The absence of carbon-related diffraction peaks at ∼25° can be attributed to both the minimal presence and the low diffraction intensity of rGO.^39^ Comparative diffraction spectra of CuO-BTC/rGO, graphene oxide (GO), and rGO substantiated the reduction of GO to rGO, facilitated by solvothermal treatment (Figure S2a,b). A distinct peak at ∼25° for 10 wt % and above CuO/rGO heterostructures (Figure 1a), denoted the graphene structure’s disordered layer predominance, likely influenced by the differential rGO concentration, similar results were observed in the literature.^39,40^

#### Raman Analysis

Raman spectroscopy serves as a quintessential instrument for delving into the internal structural nuances of carbon-based composite materials. As illustrated in Figure 1b, the Raman spectral analysis encompassed pure CuO, GO, rGO, and CuO/rGO heterostructures with varying weight percentages. The characteristic D and G bands of carbon were discernible at approximately 1364 and 1605 cm^–1^, respectively, across the spectra of GO, rGO, and CuO/rGO amalgamations (Figure 1b). Noteworthily, the *I*_D_/*I*_G_ ratio, a quantitative marker of defect density within the Raman spectra of Cu/rGO, was observed to surpass that of pristine GO. This elevation signifies the reduction of GO to rGO via solvothermal processing, as evidenced in Table S1. The augmented *I*_D_/*I*_G_ ratios within both composite formulations hint at an enhanced defect density, potentially stemming from the mild reducing effect of functional groups and the possible compromise of the sp^2^ bonding network induced by the chemical interfacing between rGO and Cu. The *I*_D_/*I*_G_ ratio for the solvothermally reduced rGO notably exceeded that of GO, indicating extensive damage to the carbon layer’s crystal structure during the reduction process. This structural compromise fostered a proliferation of defects within the sp^2^ lattice, elevating the structural disorder within the graphene matrix. The strategic incorporation of specific CuO concentrations into graphene, notably at 1, 5, 10, and 20 wt %, facilitated the amelioration of graphene’s imperfections, as evidenced by a diminution in the *I*_D_/*I*_G_ ratio. The Raman spectra delineated three distinct vibrational modes for both pure CuO and CuO/rGO-based heterostructures at approximately 298, 349, and 637 cm^–1^, attributed to the Cu–O bond vibrations within the monoclinic CuO phase. An observable decrement in the vibrational frequencies’ intensity, correlating with elevated rGO concentrations, suggests an augmented coverage of CuO nanoparticles by rGO sheets. The A_1g_ peak exhibited superior intensity relative to those of the B_1g_ and B_2g_ bands, indicating a pronounced crystallization of CuO, as corroborated by XRD analysis. This observation is in consonance with extant scholarly literature, thereby validating the experimental findings.^41^

#### X-ray Photoelectron Spectroscopy Analysis

Utilizing X-ray photoelectron spectroscopy (XPS), the valence states and surface compositions of the synthesized samples were meticulously examined. Presented in Figure 1c–e are the spectra for C 1s, O 1s, and Cu 2p corresponding to pure CuO, rGO, and the CuO/rGO composites designated as CuO/rGO-1, 5, 10, and 20. A comprehensive survey scan across all samples revealed the absence of impurities, as depicted in Figure S3. The calibration of all spectra was performed using the standard C 1s peak at 284.6 eV as a reference point. In Figure 1c, the deconvoluted C 1s spectra for both pure CuO and the CuO/rGO composites are displayed, illustrating peaks around ∼284.5, ∼286.8, and ∼288.9 eV. These peaks are indicative of the C–C, C–OH, and C=O groups, respectively, signifying the diverse carbonaceous environments present within the samples.^42^ The CuO/rGO samples showed a decrease in the intensity of oxygen-containing groups compared with pristine rGO, indicating changes in surface chemistry following the hydrothermal treatment. Furthermore, Figure 1d presents a comparison of the O 1s XPS spectra for rGO, pure CuO, and CuO/rGO. The pure CuO, rGO, and CuO/rGO-1 spectra are deconvoluted into three distinct components representing O-related species centered at 533.8, 532.3, and 530.9 eV attributed to C=O, C–OH, and O=C–OH, for rGO and centered at 531.8, 530.7, 529.3 attribute to C–O, O–Cu, and C=O for CuO and CuO/rGO-1.^43,44^ Whereas the higher content heterostructures show distinct components centered at 531.6, 530.29, 529.41, and 528.7 attributed to the C–O, O–Cu, O=C–OH, and C=O respectively. A noteworthy trend is the gradual diminution of oxygen vacancies with increasing graphene content, reaching a minimum at CuO/rGO-20. This trend suggests that the incorporation of graphene layers may influence the oxygen vacancy concentration, with the CuO/rGO samples exhibiting a higher prevalence of these vacancies than that of pure CuO. The increase in oxygen vacancies within the CuO/rGO heterostructures, as opposed to pristine CuO, is likely a consequence of the synergistic effects of rGO integration and the ensuing surface defect generation during the hydrothermal synthesis process. The enhanced gas sensing capabilities of the CuO/rGO heterostructures over pure CuO are ostensibly linked to the increased oxygen vacancy concentration within the former. This statement is further corroborated by the Cu 2p XPS spectra depicted in Figure 1e, where both CuO and CuO/rGO samples manifest the characteristic spectra of CuO. The binding energies identified at 933.2 eV for Cu 2p_3/2_ and 954.6 eV for Cu 2p_1/2_ are indicative of Cu(II) ions.^45^ Additionally, satellite peaks at 943.0 and 962.6 eV affirm the existence of unfilled Cu 3d9 orbitals, reflective of Cu^2+^ in a paramagnetic chemical state.

### Microstructural Analysis

#### Surface Morphology

**Figure 2:**
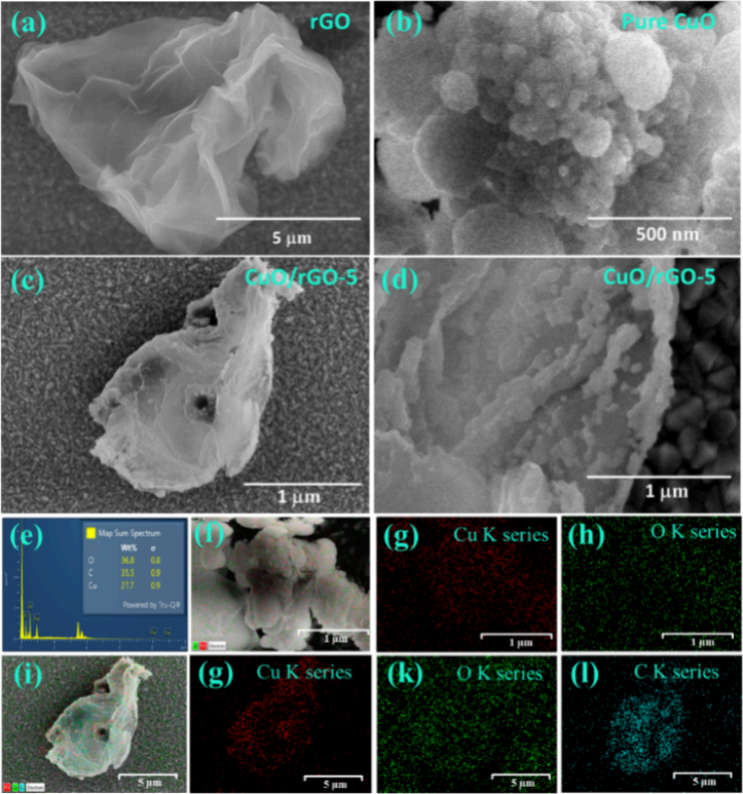
Field emission micrograph of (a) rGO, (b) pure CuO, (c,d) CuO/rGO-5 heterostructures, and (e) EDS elemental analysis of pure CuO (inset: EDS elemental spectra of CuO/rGO-5), (f–h) elemental mapping of Cu & O, and (i–l) CuO/rGO-5 elemental mapping of Cu, O, & C.

The surface morphology of synthesized pure CuO, rGO, and the CuO/rGO heterostructure with 5% rGO content (CuO/rGO-5) was analyzed using field emission scanning electron microscopy (FE-SEM). This analysis highlighted the unique structural characteristics of each material. Figure 2a shows the wrinkled, sheet-like structure of rGO, featuring visible 3–4 layers and slender edges. Figure 2b displays spherical CuO nanoparticles with a uniform size distribution. Figure 2c,d demonstrate a homogeneous dispersion of CuO nanoparticles across the rGO sheets. This uniform distribution potentially enhances the gas sensing properties of the composite by increasing both the active surface area and the number of heterojunctions between CuO and rGO. Figure 2e depicts the elemental compositions of CuO/rGO, focusing on elements such as Copper (Cu), Oxygen (O), and Carbon (C). For comparison, the elemental compositions of pure CuO are depicted in Figure S4. The energy dispersive spectroscopy (EDS) mapping, detailed in Figure 2f–l, provides deeper insight into the elemental distribution within the samples. The current elemental analysis of pure CuO and CuO/rGO heterostructures confirms that the prepared samples are free from impurities.

**Figure 3:**
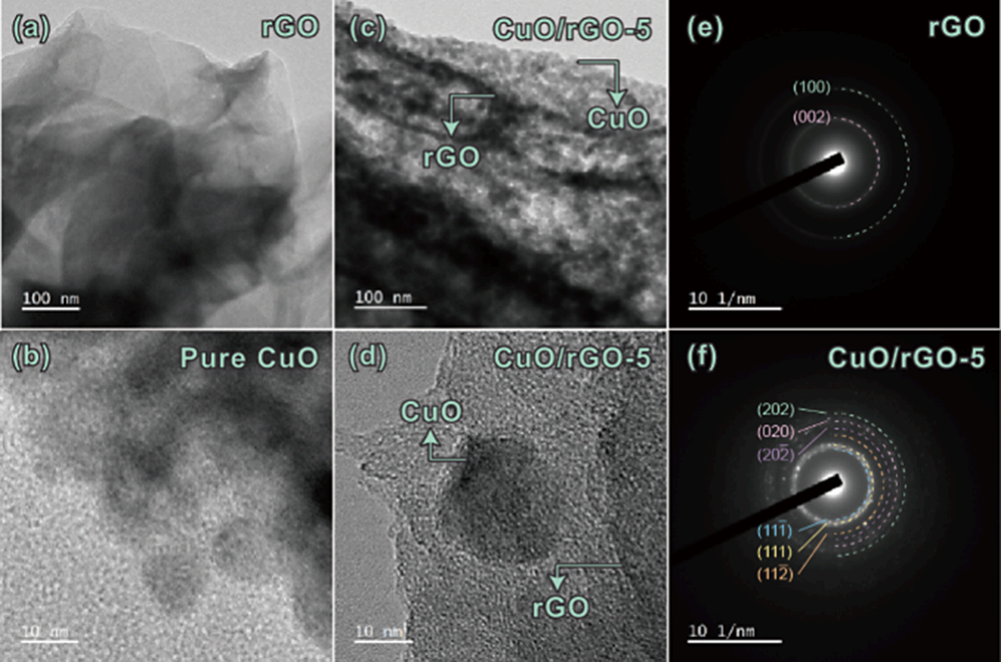
TEM micrograph of (a) rGO, (b) pure CuO, (c,d) TEM and HR-TEM of CuO/rGO-5 heterostructures, (e) SAED of rGO, and (f) SAED of CuO/rGO-5 heterostructures.

Transmission electron microscopy (TEM) and high-resolution TEM (HR-TEM) analyses of rGO, pure CuO, and CuO/rGO-5 heterostructures are presented in Figure 3. The TEM images of rGO show typical interconnected, sheet-like structures with a wrinkled surface and a clumped appearance as seen in Figure 3a. The low-loading rGO in the CuO/rGO-1 heterostructures displays agglomerates of very small, scattered nanoparticles, averaging 35 nm in size (Figure S5). In contrast, the higher rGO-based loading in the CuO/rGO-5 sample reveals a homogeneous distribution of smaller nanoparticles, averaging 30 nm, as shown in Figure 3c. Additionally, Figure 3d illustrates nanoparticles averaging 25 nm wrapped in a graphene sheet, alongside numerous tiny nanoparticles embedded within the graphene layers (Figure 3c). This dispersion highlights the ability of graphene sheets to support the growth of CuO nanoparticles in various shapes and sizes, fostering robust interconnected surfaces that develop through a synthesis process influenced by the dynamics of growth and dissolution. The selected area electron diffraction (SAED) patterns of rGO and CuO/rGO-5, depicted in Figure 3e,f, show rGO sheets as poly nanocrystalline in nature. The fringe spacing between the lattices measures 3.5 and 2.7 Å (Figure 3e), matching the *d*-spacing of the (002) and (100) reflection planes, respectively. Additionally, the continuous and diffused rings are indexed to the (111̅), (111), (112̅), (202̅), (020), and (202) planes of the monoclinic phase of CuO in the CuO/rGO-5 heterostructure.

### Gas Sensing Performance

**Figure 4:**
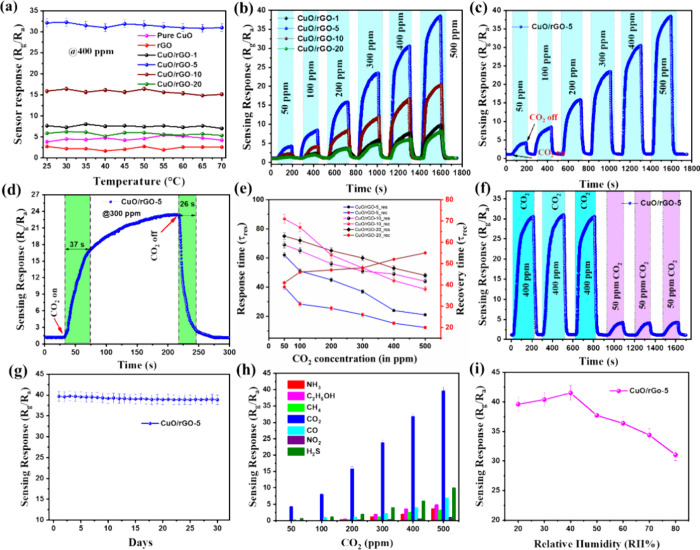
(a) Temperature-dependent CO_2_ sensing response of all samples to 400 ppm of CO_2_, (b) dynamic CO_2_ sensing response-recovery curve of CuO/rGO-1, 5, 10, and 20, (c) dynamic resistance variation curves of the CuO/rGO-5 for different CO_2_ content, (d) response and recovery curve of CuO/rGO-5 at 300 ppm of CO_2_ content, (e) transient response/recovery curves of CuO/rGO-5, 10, and 20 heterostructures for different content of CO_2_, (f) dynamic resistance variation curves of the CuO/rGO-5 at 400 and 50 ppm of CO_2_, (g) long-term stability curve of the CuO/rGO-5 at 500 ppm of CO_2_, (h) Selectivity bar diagram of all the samples for different analyte gases, (i) Humidity effect on the prepared CuO/rGO-5 CO_2_ sensor.

The critical role of operating temperature in the efficacy of gas sensing, particularly for metal-oxide semiconductor-based chemiresistors, is well-documented.^46,47^ These sensors are known to exhibit temperature-dependent performance characteristics, including variations in response and recovery times. Consequently, an exploration was conducted to ascertain the optimal temperature range for CO_2_ gas sensing, focusing on a span from 25 to 70 °C, with incremental adjustments of 5 °C (gas sensing experimental details are described in the Supplementary Information, a gas sensing experimental schematic is depicted in Figure S6). This investigation was illustrated in Figure 4a, which delineated the sensor response to 400 ppm of CO_2_ across the specified temperature range for p-type heterostructure sensors. The findings highlighted a plateau in the sensor response commencing at 25 °C, establishing this as the optimal operating temperature for subsequent evaluations.

A peculiar decrease in the sensor response at 45 °C was observed, suggesting a modification in the CO_2_ gas adsorption mechanisms. Further sensitivity tests at room temperature toward CO_2_ concentrations ranging from 50 to 500 ppm were conducted on samples comprising pure CuO, rGO, and CuO/rGO heterostructures (with variations of 1, 5, 10, and 20 wt %) deposited over Interdigitated Electrodes (IDE). The dynamic response-recovery behavior of these samples to CO_2_ is depicted in Figure S7. Notably, pure CuO sensors exhibited incremental responses to increasing CO_2_ concentrations with a maximum response of 8.7% at 500 ppm. Conversely, pure rGO-based sensors showed negligible sensitivity to CO_2_ at lower concentrations. The incorporation of rGO into CuO significantly enhanced sensor responses, with the CuO/rGO nanocomposites demonstrating responses up to 39.6% at higher CO_2_ levels (i.e., 500 ppm), marking a 10-fold increase in sensitivity compared to pure CuO sensors. This enhancement in sensor performance was most pronounced with a 5 wt % addition of rGO, beyond which the sensor efficiency gradually declined. The highest sensitivity of CuO/rGO-5 can be attributed to one of the reasons is 5 wt % rGO is an optimized concentration for creating the maximum number of heterojunctions between the metal-oxide and rGO. Additionally, higher concentrations of rGO may obscure the active surface area, leading to diminished sensing responses. This effect has been similarly observed in other rGO-based materials documented in the literature.^48,49^

The superior performance of CuO/rGO-5 heterostructures at room temperature underscores the potential of rGO incorporation in boosting gas sensing capabilities. Figures S7 further elucidate the sensors’ dynamic sensing responses, illustrating a consistent rise in resistance with increasing CO_2_ levels. This resistance trend, indicative of the sensors’ operational characteristics, was systematically reported, affirming the CuO/rGO heterostructures’ suitability for CO_2_ detection in environmental and indoor settings. To gain a deeper understanding of the sensing behavior of the prepared sensors, we have included additional low-range sensor response curves for CuO/rGO-1, CuO/rGO-5, CuO/rGO-10, and CuO/rGO-20 samples exposed to 100 ppm of CO_2_ in Figure S10a-d. These curves clearly illustrate the changes in response during gas exposure, highlighting both the increase and subsequent decrease in resistance. The sensing parameters of the prepared samples, tested under various CO_2_ concentrations, are detailed in Table S2. Additionally, a comparative analysis of the CO_2_ sensing performance related to our study is provided in Table S3. This analysis shows that the operational temperature of the CuO/rGO nanocomposite-based sensors is significantly lower compared with previously reported CO_2_ gas sensors. For instance, Bhowmick et al.^50^ reported a CuO/ZnO bilayer thin film for CO_2_ detection that exhibited a higher sensor response but required an operating temperature of 375 °C. In contrast, our current CO_2_ sensor achieves a comparable sensing response at room temperature. Thus, Table S3 briefly highlights the innovative advantage of this research in the development of efficient, low-temperature gas sensing technologies compared to recent reports.

The dynamics of response and recovery times are one of the key factors for assessing the effectiveness of gas sensors. Figure 4d depicts the response and recovery curve of the CuO/rGO-5 sensor to a 300 ppm of CO_2_ concentration, with fast response and recovery times of 37 and 26 s, respectively. Further investigation into the response and recovery behaviors of CuO/rGO-5, CuO/rGO-10, and CuO/rGO-20 samples at various CO_2_ concentrations is documented in Figure 4e, while the results for other samples are presented in Figure S8 and Table S2. Figure 4e concisely highlights the response and recovery times of the CuO/rGO material under ambient conditions, emphasizing its operational efficiency. The sensor’s detection capabilities were evaluated, determining a Limit of Detection (LOD) for the present CO_2_ sensor, based on a linear response-concentration relationship (Figure S9). The calculated LOD for the prepared CuO/rGO-5 sensor was 2 ppm. The LOD calculation, derived using the formula 3σ/m, indicates a sensitivity that significantly exceeds both the Threshold Limit Values (TLVs) for CO_2_ and typical human exhalation levels, marking a notable improvement in detection accuracy. The repeatability and stability of the CuO/rGO-5 sensors were confirmed through multiple sensing cycles, as depicted in Figure 4f. Consistent gas responses across three consecutive tests for 400 and 50 ppm of CO_2_ concentrations highlight the sensor’s excellent repeatability. A 30-day long-term stability test of the prepared CuO/rGO-5 showed minimal variation in the sensor response, within an experimental error margin of less than 1.9%, which is attributed to the stable oxidation states of the constituent layers, as illustrated in Figure 4g.

The selectivity of the CuO/rGO-5 sensor was tested by exposing it to various interfering gases, including CO, NH_3_, H_2_S, NO_2_, CH_4_, and CH_3_OH, each at a concentration of 500 ppm and at room temperature. Figure 4h demonstrates the CuO/rGO-5 sensor’s pronounced selectivity toward CO_2_, a trait attributed to factors such as bond energy, gas molecular mass, and the specific morphology and crystal orientation of the gas-sensitive material. The prepared CuO/rGO-5 sensor exhibited significantly higher sensitivity to CO_2_ over other gases like CO, H_2_S, NO_2_, CH_4_, C_2_H_5_OH, and NH_3_, and exhibited no response to N_2_, highlighting its exceptional specificity. We also conducted additional experiments to examine the sensor response of CuO/rGO-5 samples to N_2_, O_2_, and a 50:50 mixture of the two gases at a concentration of 500 ppm (shown in Figure S11a–c). The findings revealed negligible sensitivity to both gases under the tested conditions, emphasizing the sensor’s selectivity toward the target analytes (Figure S11a,b). However, a very low response was observed for the N_2_/O_2_ mixture (Figure S11c). The influence of humidity on gas sensing was also meticulously investigated, acknowledging its importance in the context of semiconducting sensor layers. Resistance measurements across a humidity range of 20–80 RH% revealed the nuanced impact of moisture on CO_2_ sensing, as depicted in Figure 4i. The interaction between water molecules and ionized oxygen species, leading to electron donation and the formation of hydroxyl groups, elucidates the observed decrease in the sensor response at higher humidity levels. This suggests that while OH groups facilitate CO_2_ adsorption on the metal oxide surface, effective CO_2_ sensing also necessitates sites capable of electron donation. However, as humidity levels rise, the availability of such oxygen electron donors diminishes, indicating a need for an optimal humidity level for peak sensor performance, identified at 40% RH for 500 ppm of CO_2_. This comprehensive examination of the CuO/rGO sensor’s performance across various parameters response and recovery times, LOD, repeatability, selectivity, and humidity influence underscores its potential for enhanced CO_2_ detection. Future research will delve into further optimizing sensor performance, particularly in reducing humidity cross-sensitivity, to refine detection limits and layer modifications.

### Sensing Mechanisms

**Figure 5:**
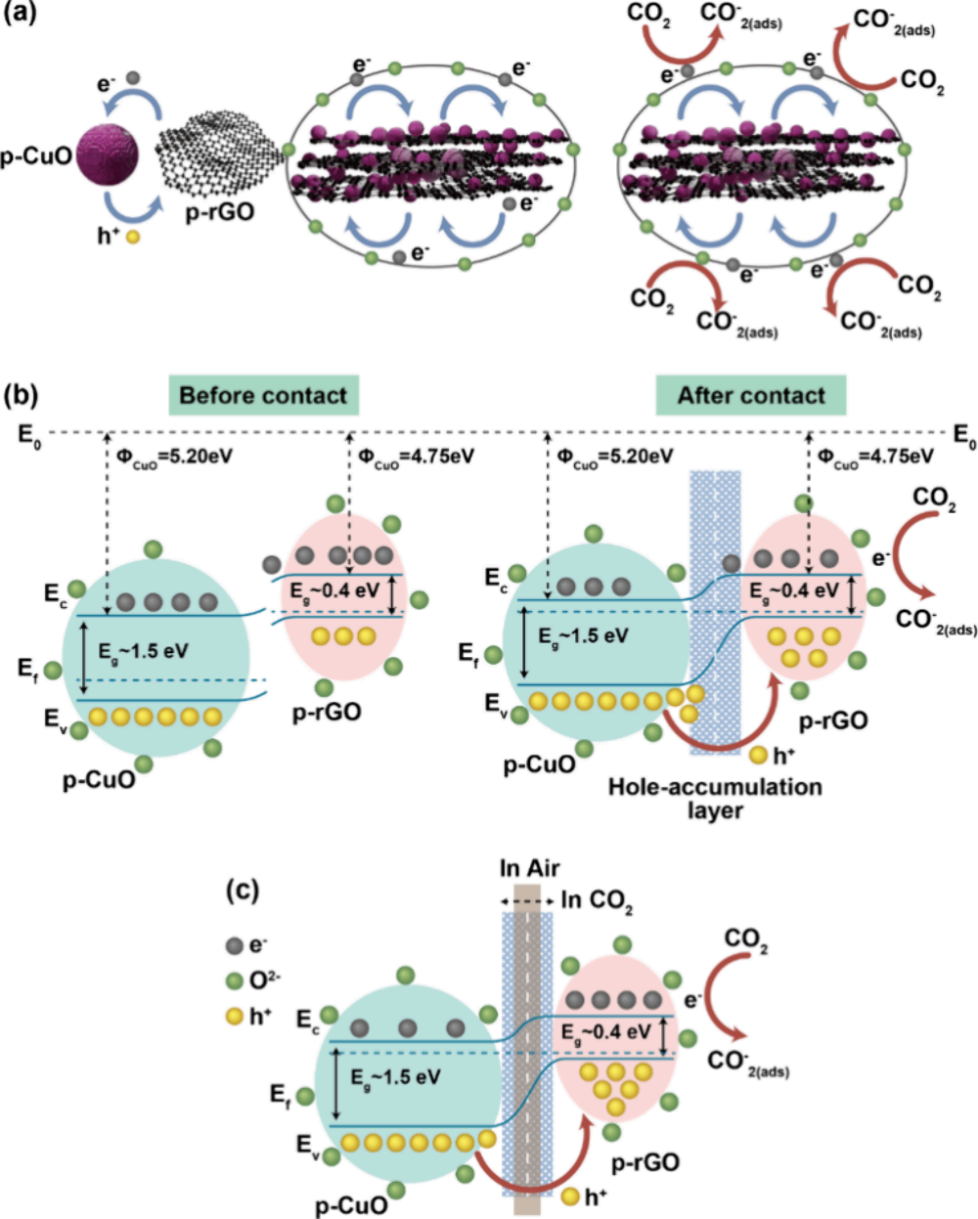
(a) Proposed schematic representation of the CO_2_ gas sensing mechanism, (b) illustration of the formation of the p-p heterojunction of CuO and rGO, and (c) energy band diagram of CuO NPs combined with rGO.

The functioning of a p-p-type heterojunction sensor is driven by the adsorption/desorption processes characteristic of a p-p-type metal oxide-semiconductor. As oxygen molecules are absorbed onto the grain surfaces, electrons are drawn from the valence band to these oxygen molecules. This electron transfer results in the formation of a hole-accumulation layer (HAL) on the surface.^51,52^ The gas sensing mechanism of the present p-p type CuO/rGO heterostructures is comprehensively explained through the framework of conventional adsorption theory, as depicted in Figure 5. Prior research underscores that both pure CuO and rGO inherently possess p-type semiconductor properties.^53,54^ This foundational premise guides the explanation that, upon exposure to air, oxygen molecules engage with these materials by capturing electrons, leading to the formation of chemically adsorbed oxygen ions on the material’s surface, as illustrated in Figure 5a. Here, the monoclinic structure of CuO, characterized by its dangling bonds, exhibits a high catalytic activity for O_2_ adsorption on its surface. PXRD reveals strong (111̅) and (111) peaks in the prepared polycrystalline CuO nanostructure, indicating a prominent presence of these planes on the CuO surface. This structure facilitates the formation of a large hole accumulation region on the CuO surface when exposed to an open atmosphere. Under ambient conditions, CO_2_ serves as a reducing agent. The oxidization of CO_2_ occurs via the negatively charged oxygen ions adsorbed on the surface, leading to electron release back into the material and the formation of CO_2_^–^_(ads)_.^55^ Consequently, as electrons and holes recombine, the hole concentration near the surface decreases, which in turn increases the device’s resistance.

In other words, the cornerstone of these heterostructures’ detection capability lies in the interaction between gas molecules and the adsorbed oxygen ions, which precipitates a change in the sensor’s resistance. This interaction is underpinned by the chemical processes delineated in eqs 1 and 2, where oxygen molecules in their gaseous state (O_2__(gas)_) become adsorbed onto the surface (O_2__(ads)_) and subsequently capture an electron to form O^2–^ ions:
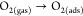
1
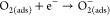
2

The gas sensing performance of CuO/rGO heterostructures is significantly enhanced by the formation of heterojunction interfaces between rGO and CuO. The CO_2_ sensing mechanism in these heterostructures is influenced by the numerous small heterojunctions and the inherent chemical properties of both rGO and CuO. An increase in reactive sites, due to the rGO being decorated on the CuO, facilitates greater gas adsorption on the device’s surface, resulting in an improved response to gas detection. Figure 5b illustrates the schematic representation of the p-p heterojunction formation, both pre- and postgas exposure. In these heterojunctions, the sensing response is primarily attributed to the CuO surface covered with rGO nanosheets, as depicted in Figure 5b. This is because there is a relatively smaller quantity of rGO on the CuO surface. The interplay between CuO nanoparticles and rGO, driven by their respective work functions CuO at approximately 4.75 eV and rGO around 5.20 eV facilitates the migration of holes from CuO to rGO and electrons in the converse direction until an equilibrium is established at both Fermi levels.

This p-p heterojunction formation, alongside modifications in the thickness of the hole accumulation layer at the heterojunction boundary, significantly contributes to the sensor’s enhanced detecting capabilities, as showcased in Figure 5c. The charge transfer across the p-p junction, propelled by inherent electric fields, engenders a depletion layer, thereby augmenting the sensor’s resistance and establishing potential barriers. In the presence of air, electrons ensnared by adsorbed oxygen augment the availability of holes near the surface. When encountering reducing CO_2_ gas, the chemisorbed CO_2_ escalates the number of available holes near the material’s surface, thereby thickening the hole accumulation layer and further elevating resistance, as elucidated in Figure 5c.

The integration of rGO into CuO not only diminishes the electrical resistance when aired but also amplifies it upon CO_2_ exposure. This phenomenon is attributed to the reaction between the O^2–^ ions and CO_2_, leading to decomposition into CO_2_^–^ and the release of electrons, responsible for the resistance increase, as outlined in eqs 3 and 4:
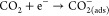
3
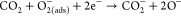
4

This process, wherein CO_2_ gas molecule removal regenerates the charge carriers within the accumulation layer, culminates in a sensor resistance reduction, encapsulating the intricate interplay of reactions that underpin the gas sensing mechanism of the CuO/rGO heterostructures.

## Conclusions

In conclusion, our study successfully synthesized CuO/rGO heterostructures derived from an MOF template using the solvothermal method. This investigation highlighted that the nanocomposites, characterized by the direct attachment of CuO nanoparticles to the 2D surface of graphene, undergo significant morphological and structural changes compared with their pure CuO and rGO counterparts. The p-p-type CuO/rGO heterostructures, especially those with a 5 wt % rGO composition, showcased superior gas sensing performance at ambient temperature. Notably, the sensing response of rGO-CuO-5 to 500 ppm of CO_2_ was 39.6, which is 10-fold greater than that of pure CuO and 8-fold higher than that of rGO alone. Furthermore, the LOD for CO_2_ was determined to be 2 ppm, based on linear regression analysis of the sensor response data.

The findings from density functional theory (DFT) calculations further elucidated the formation of p-p heterojunctions, underpinning the sensing mechanism proposed based on theoretical insights. The observed higher adsorption energy of the p-p heterostructure compared with other samples underscores its potential for developing a highly selective CO_2_ gas sensor. This work not only advances our understanding of CuO/rGO heterostructures in gas sensing applications but also opens up new avenues for the design and optimization of sensitive and selective gas sensors for environmental monitoring and other critical applications. Such advancements hold great potential for improving indoor air quality and enhancing agricultural productivity, directly benefiting public health and sustainability.

## Supporting Information Available

Experimental details on the solvothermal preparation of CuO-BTC and CuO/rGO, along with their schematic diagrams (Figure S1); detailed characterization techniques; XRD spectra for Cu(BTC), Cu(BTC)_rGO, GO, and rGO (Figure S2a,b); *I*_D_/*I*_G_ ratios for GO, rGO, pure CuO, and CuO/rGO heterostructures at varying concentrations (CuO/rGO-1, CuO/rGO-5, CuO/rGO-10, CuO/rGO-20) (Table S1); survey scan XPS spectra for rGO, CuO, and the aforementioned CuO/rGO heterostructures (Figure S3); elemental compositions of pure CuO and rGO (Figure S4); TEM micrograph of CuO/rGO-1 (Figure S5); details on device fabrication and gas sensing measurements, including a schematic diagram of the gas sensing setup (Figure S6); dynamic resistance variation curves for the CuO/rGO series (Figure S7); dynamic response/recovery curves of pure CuO, rGO, and CuO/rGO-1 for different content of CO_2_ (Figure S8); sensor responses at various CO_2_ concentrations ranging from 50 to 500 ppm (Figure S9 and Table S2); different low-curve dynamic response data and selectivity study for N_2_/O_2_ (Figures S10 and S11); comparison of key CO_2_ sensor parameters from earlier report (Table S3); and computational details and details analysis for theoretical calculations was described in details, with banddiagram and optimized structures in Figure S11 and Table S4 (PDF)
