# Supplementary material for: Exploring MOF-Derived CuO/rGO Heterostructures for Highly Efficient Room Temperature CO2 Sensors
Source: ACS Sens. 2024 Sep 18;9(11):5856–65. doi: 10.1021/acssensors.4c01397 (PMC11590104; doi:10.1021/acssensors.4c01397)
Supplement: Supplementary file 1 — se4c01397_si_001.pdf [file se4c01397_si_001.pdf]

Supporting Information

Exploring MOF derived CuO/rGO heterostructures for highly efficient room temperature CO_2 sensor

Toton Haldar[a], Jia-Wei Shiu[b], Ren-Xuan Yang[c], Wei-Qi Wang[b], Hsin-Ting Wu[b], Hsu-I Mao[b], Chin-Wen Chen[b],*, and Chi-Hua Yu[a*]

[a] *Department of Engineering Science, National Cheng Kung University, Tainan 701401, Taiwan*

[b] *Department of Molecular Science and Engineering, National Taipei University of Technology, Taipei 106344, Taiwan*

[c]*Institute of Environmental Engineering and Management, National Taipei University of Technology, Taipei 106344, Taiwan*

*Corresponding author. E-mail: cwchen@ntut.edu.tw and jonnyyu@gs.ncku.edu.tw

Experimental Section

All the chemicals were of analytical grade and used as received without purification. Copper (II) nitrate trihydrate ($Cu(NO_3)_2 \cdot 3H_2O$ (AR, 99%), lauric acid ($C_{12}H_{24}O_2$) benzene-1,3,5-tricarboxylic acid (btc, AR, 99%), 1-butanol (AR, 99%) were purchased from Sigma Aldrich, USA. Graphene oxide was purchased from Taiwan Carbon Materials Corp., Taiwan, ROC.

Preparation of CuO/rGO nanocomposites. In this report, p-type CuO combined with the reduced graphene oxide was synthesized from the MOF template by a single-step solvothermal method (**Figure S1**). The synthesis of p-p type CuO/rGO heterostructures began with the preparation of high-crystalline metal-organic framework (MOF) powders, namely copper benzene-1, 3, 5-tricarboxylate polyhedron ([Cu3(btc)2]n), using the solvothermal method.[1,2] Subsequently, the MOF powders were subjected to thermal decomposition to obtain pure copper oxide (CuO) and a composite of CuO with reduced graphene oxide (CuO/rGO).

In this experiment, a total of 205 mg of copper (II) nitrate trihydrate (AR, 99%), 6.102 g of lauric acid, and 100 mg of benzene-1,3,5-tricarboxylic acid (btc, AR, 99%) were dissolved in 60 mL of butanol (AR, 99%). Following a vigorous stirring period of 10 minutes, the necessary quantity of graphene oxide (1, 5, 10, and 20 wt%) was dispersed onto the MOF solution by a combination of ultrasonication and magnetic stirring, alternating between the two methods for 30 minutes. Subsequently, the GO-MOF solution was transferred into a Teflon-lined stainless-steel autoclave with a volume of 160 ml. The autoclave was heated to a temperature of 140 °C and held at this temperature for 3 hours before being cooled in ambient air. The precipitates were separated using the process of centrifugation, followed by multiple washes using ethanol and DI water to eliminate any potential residues. Subsequently, the Cu-btc/rGO-MOF precipitates were vacuum-dried overnight. The CuO/rGO heterostructure was synthesized in the last phase through the thermal degradation of $[Cu_3(btc)_2]$n-GO MOF in an air environment at a temperature of 300 °C for 1 hour. The pure copper (II) oxide (CuO) was prepared using a similar process involving the thermal decomposition of $[Cu3(btc)_2]$n in an air environment at a temperature of 300 °C for 1 hour. The weight ratios of the GO and CuO p-p type heterostructures gas sensor were 1, 5, 10, and 20 wt%,

abbreviated as pure CuO, CuO/rGO-1, CuO/rGO-5, CuO/rGO-10, and CuO/rGO -20, respectively.

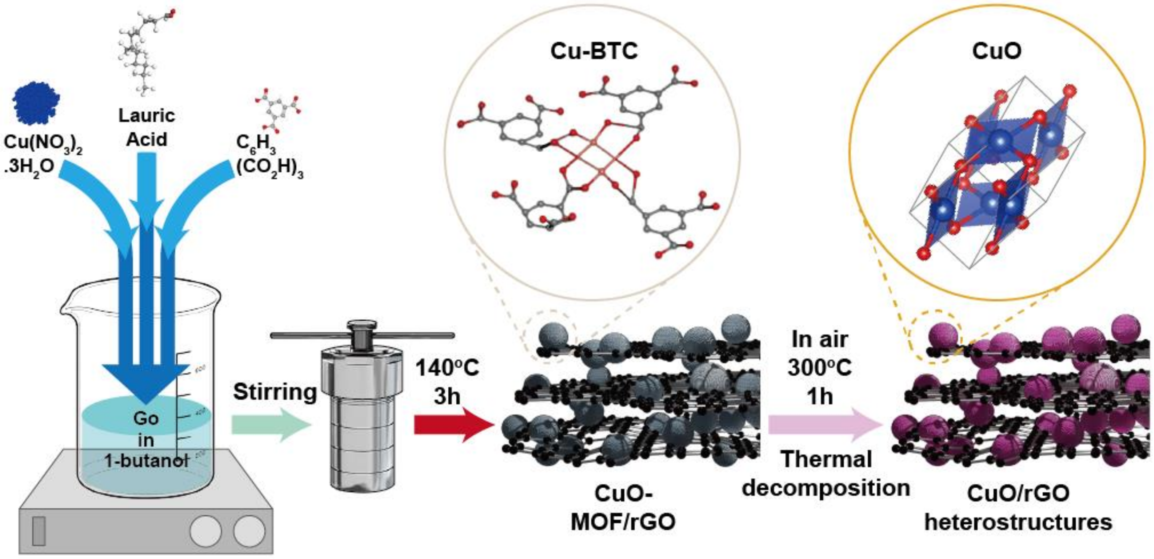

Figure S1 *Schematic representation of one-pot synthesis of CuO/rGO from MOF template.*

Material characterization. The X-ray diffraction analysis of the annealed pure CuO, GO, rGO, and CuO/rGO heterostructures samples was conducted using a PW-XRD instrument (X'pert-PRO, PANalytical, Netherlands) with CuKα radiation (λ = 1.5418 Å). Raman spectroscopy with an excitation wavelength of 532.10 nm characterized the carbon content of the CuO/rGO heterostructures. The morphologies and microstructure analysis of the prepared pure and composite samples were carried out by a field emission scanning electron microscope (FE-SEM, HITACHI, Regulus 8100) equipped with an energy dispersive X-ray spectroscope (EDS). Additionally, a transmission electron microscope (TEM, JEM-2100F (HR), JEOL Ltd, Japan) with selected-area electron diffraction (SAED) was employed for high-resolution analysis. The surface composition and chemical oxidation state were analyzed using an X-ray photoelectron spectroscope (JPS 9030, JEOL Ltd) equipped with a monochromatic AlKα X-ray source (energy = 1486.6 eV).

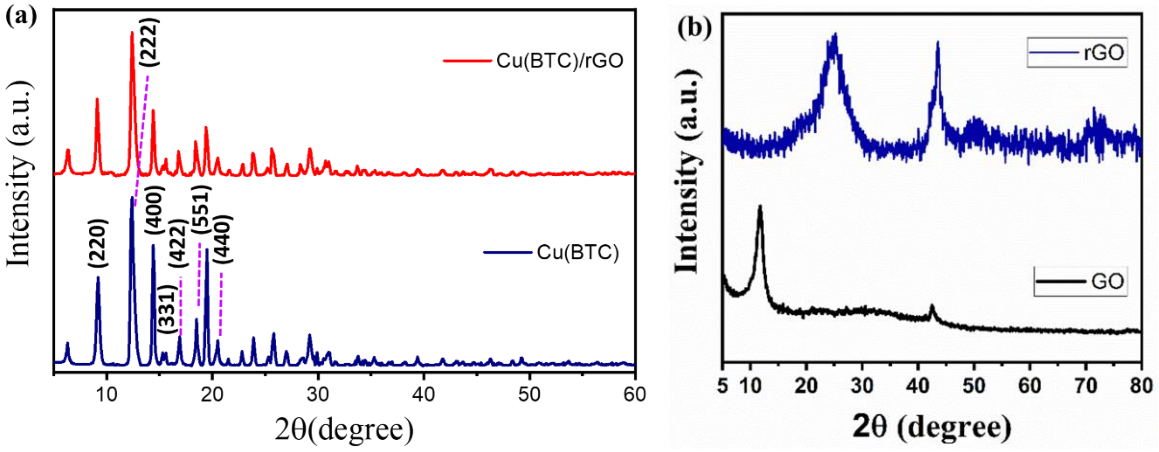

Figure S2 *a) XRD spectra of Cu(BTC) and Cu(BTC)/rGO, b) XRD spectra of GO and rGO*

Table S1 *The D and G band position and I_D/I_G of GO, rGO, pure CuO, CuO/rGO-1, CuO/rGO-5, CuO/rGO-10 and CuO/rGO-20 heterostructures*

Samples	D band (cm^{-1})	G band (cm^{-1})	I_D/I_G
GO	1373	1609	0.93
rGO	1369	1598	1.01
CuO/rGO-1	1385	1613	1.03
CuO/rGO-5	1377	1608	1.04
CuO/rGO-10	1382	1611	1.09
CuO/rGO-20	1379	1613	1.13

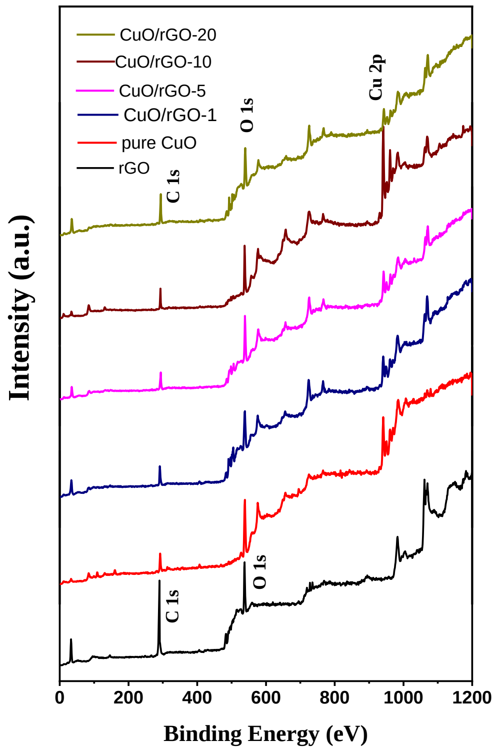

Figure S3 *Survey scan XPS spectra of rGO, CuO, CuO/rGO-1, CuO/rGO-5, CuO/rGO-10, and CuO/rGO-20*

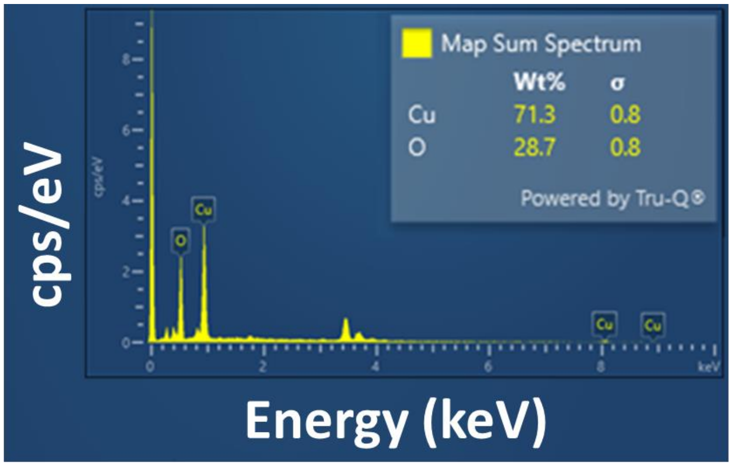

Figure S4: *EDX spectra of pure CuO derived from CuO-MOF*

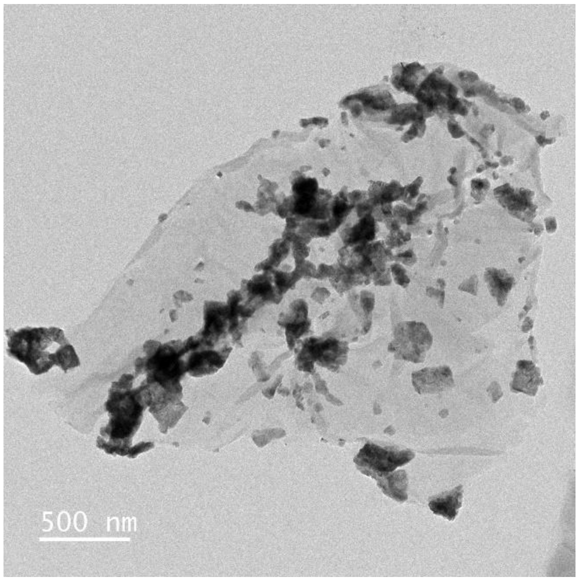

Figure S5 *TEM micrograph of CuO/rGO-1*

Device fabrication and gas sensing measurements. Figure S6 illustrates the schematic of the gas sensing experimental setup. Samples of pure CuO, rGO, and CuO/rGO heterostructures were dispersed in ethanol and sonicated for 15 minutes to achieve a homogeneous solution. The active gas sensor chip was fabricated by drop-casting 50 µl of this solution onto interdigitated Pt electrodes (IDE), followed by ethanol evaporation at 40 °C for 15 minutes. Gas sensing performance tests were conducted in a temperature-controlled chamber, as depicted in Figure S6. The device was evaluated by exposing it to various concentrations of harmful gases, ranging from 50 to 500 ppm, controlled via Mass Flow Controllers (MFC). After optimizing the operating temperature and other parameters, gas sensing performance was assessed under constant conditions. The sensor device's resistance change was recorded using a Keithley source meter (Model: 2400) with an applied potential of 1.5 V. Gas sensing response was determined by calculating the ratio of sensor resistance in test gas (R_g) to that in air (R_a).

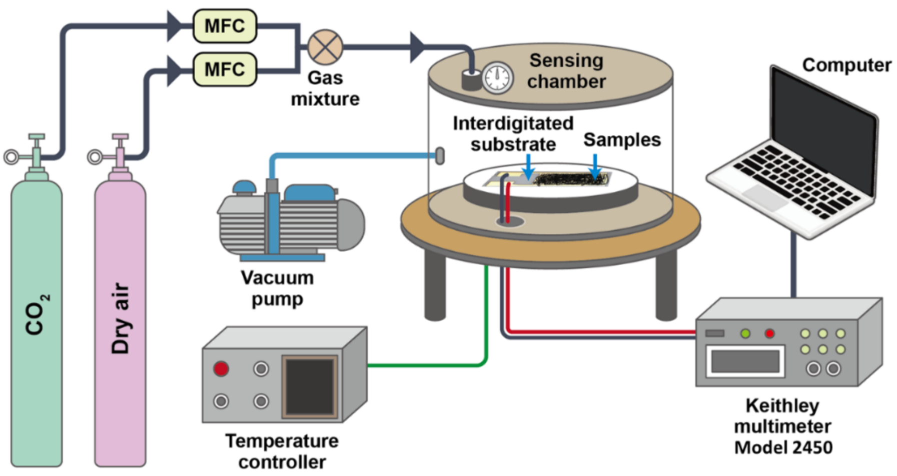

Figure S6 Schematic representation of the experimental sensing setup used to measure different gas concentrations with heterostructure sensor in real time.

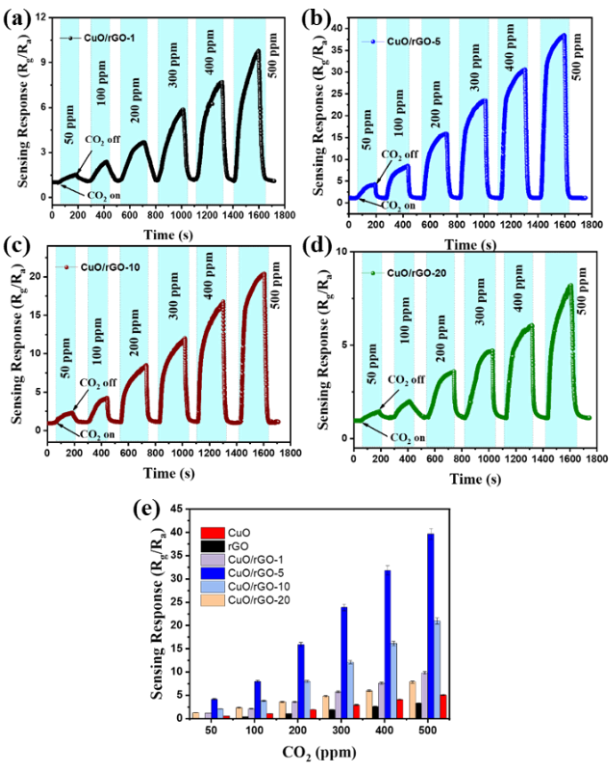

Figure S7 *a) dynamic resistance variation curves of the CuO/rGO-1, b) dynamic resistance variation curves of the CuO/rGO-5, c) dynamic resistance variation curves of the CuO/rGO-10 and d) dynamic resistance variation curves of the CuO/rGO-20, and e) Sensor response of all the prepared* CO_2 *gas sensor at different concentrations of* CO_2*.*

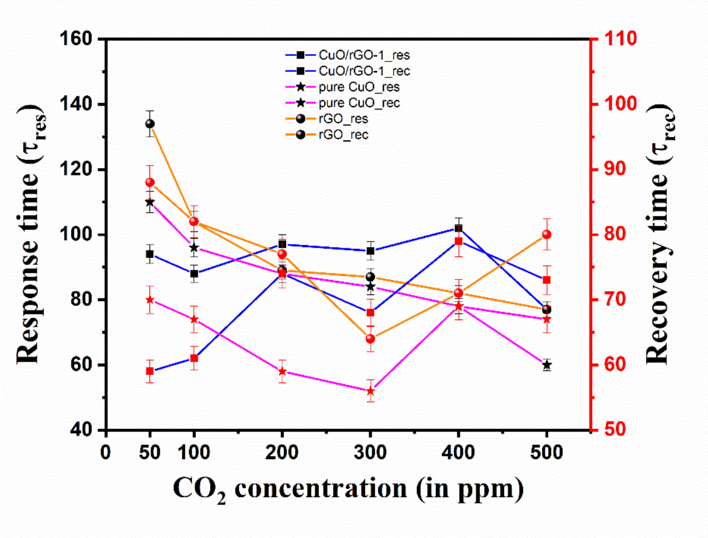

Figure S8: *Transient response/recovery curves of pure CuO, rGO, and CuO/rGO-1 heterostructures for different content of* CO_2*.*

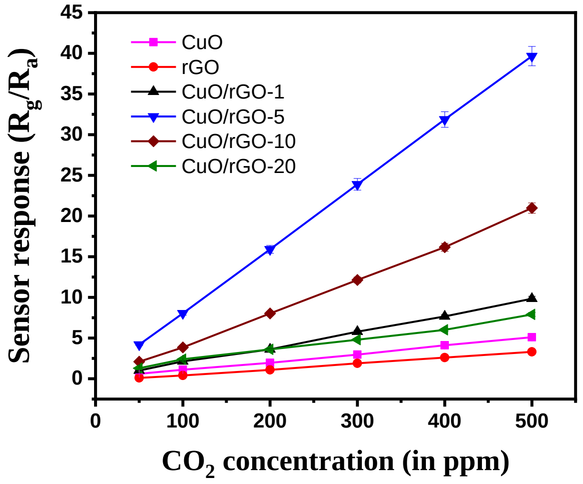

Figure S9: *Sensor response at different* CO_2 *content from 50 ppm to 500 ppm.*

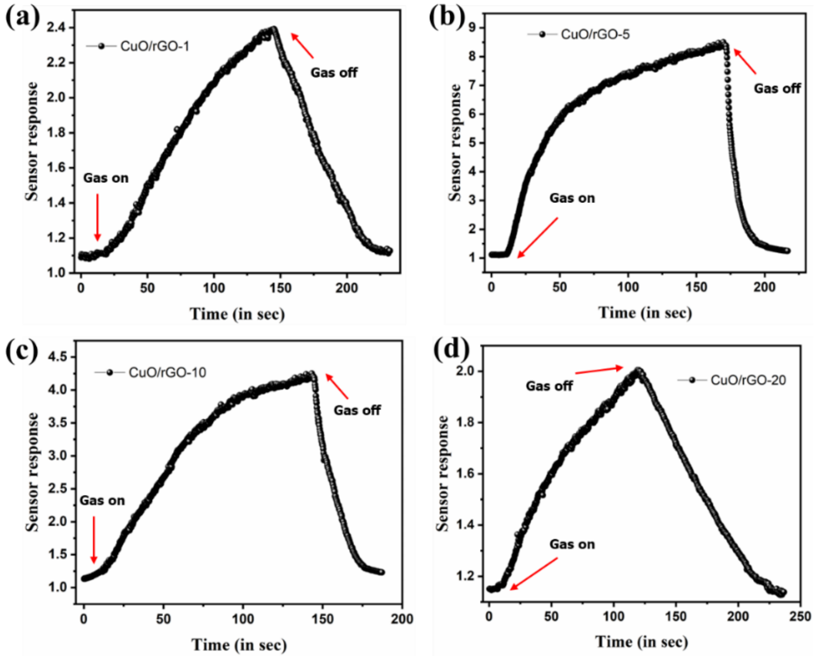

Figure S10: *Low-range dynamic sensor response curves of (a) CuO/rGO-1, (b) CuO/rGO-5, (c) CuO/rGO-10, and (d) CuO/rGO-20 samples under 100 ppm* CO_2.

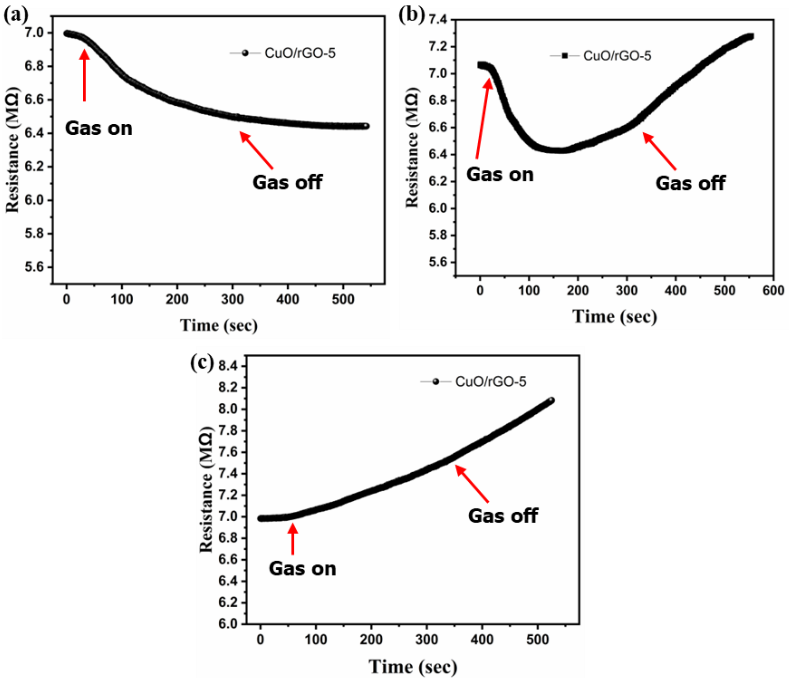

Figure S11: Dynamic sensing response of CuO/rGO-5 samples to (a) N□, (b) O□, and (c) 50:50 N□/O□ mixture at 500 ppm concentration.

Table S2 *Calculated sensing parameters: Sensing response (S), response time (τ_{res}) and recovery time (τ_{rec}) of pure CuO, rGO and CuO/rGO* heterostructures *samples.*

**Samples**	**Pure CuO**			**rGO**			**CuO/rGO-1**			**CuO/rGO-5**			**CuO/rGO-10**			**CuO/rGO-20**		
**CO_2 (in ppm)**	**S**	τ_{res}	τ_{rec}	**S**	τ_{res}	τ_{rec}	**S**	τ_{res}	τ_{rec}	**S**	τ_{res}	τ_{rec}	**S**	τ_{res}	τ_{rec}	**S**	τ_{res}	τ_{rec}
**50**	0.6	110	70	0.3	134	88	1.1	94	59	4.2	62	39	2.1	69	71	1.3	75	41
**100**	1.2	96	67	0.5	104	82	2.1	88	61	8.0	51	31	3.8	65	67	2.4	72	46
**200**	1.9	88	59	1.1	89	77	3.6	97	74	15.9	45	29	8.0	56	54	3.6	65	47
**300**	2.9	84	56	1.9	87	64	5.7	95	68	23.9	37	26	12.1	51	48	4.8	60	48
**400**	4.1	78	69	2.6	82	71	7.6	102	79	31.8	24	22	16.2	49	42	6	53	52
**500**	5.2	60	67	3.3	77	80	9.8	77	73	39.6	21	20	20.9	44	38	7.9	48	55

*S: Sensor response τ_{res}: Response time τ_{rec}: Recovery time

Table S3. Comparison of key sensor parameters from this study with reported CO_2 sensors in the literature

Sensing materials	$T_{O.P}$ (^oC)	Detection range (ppm)	Response and recovery time (τ_{res}/τ_{rec}) (in sec)	Highest Sensing response	LOD (ppm)	Selectivity	Humidity effect on the sensor device	▪ Advantages / • disadvantages of the system
**This Work**	**RT**	**50-500**	**21s/20s**	**39.6**	**2**	**High Selective to CO_2**	**Sensing performance decline at ≥ 50%RH**	▪ **Highly selective to CO_2 at room temp.** • **Low response at higher %RH**
Pd:La_2O_3[3]	300	400	80s/50s	64	NA	Highly selective to CO_2	Sensor performance declines with higher humidity.	▪ High sensitivity • High operating temperature
CuO/ZnO[4]	375	2500	44s/214s	47	NA	Highly selective to CO_2	Stable response ≥ 46 %RH	▪ Long-term stability • High operating temperature, slow recovery time
Ov-$ZnSnO_3$ NCs[5]	RT	50-400	16.8s/187.5s	14.37	3.05	Purple light illumination shows highest selectivity	Sensing response decline ≥ 40 %RH	▪ Facile to fabricate • Low stability
Graphene PEI/PEG[6]	RT	1000–5000	300s/400s	32%	NA	NA	Highest response at 60 %RH	▪ Highly reversible in N_2

								• Slower response and recovery time
Sb_2O_3/graphene[7]	RT	50	16s/22s	~0.7	NA	Highly selective to CO_2	NA	■ Low operating temperature, long-term stability<br>• Lower sensing response
Al_2O_3/ Graphene[8]	RT	100	14s/22s	10.84	NA	Highly selective to CO_2	Linear sensing response ≥ 80 %RH	■ Long-term stability, low operating temperature<br>• Slow response compared to MOS
CaO−In_2O_3[9]	230	300-5000	>1000 s/40 min	~1.8	NA	NA	NA	■ Easy to fabricate, tunable properties<br>• Higher operating temperature, slower response/recovery time
$LaFeO_3$ – SnO_2[10]	250	4000	<20 s/-	2	NA	Highly selective to CO_2	NA	■ Easy synthesis method<br>• High operating temperature, low response
rGO/NiO-In_2O_3[11]	RT	50	6s/18s	40	NA	Highly selective to CO_2	NA	■ Highly sensitive, low operating temperature

CeO2/CdS[12]	RT	1000	12/20	30	NA	Highly selective to CO_2	Sensor response is dependent on humidity, decreases with increase %RH	• Humidity effect was not studied<br>▪ Self-powered gas sensor, fast response/recovery time<br>• Low sensor response compared to MOS

LOD: limit of detection; RT: Room temperature. S: Sensitivity

Computational Details. The ground state geometries were optimized at the Density Functional Theory (DFT) level using the LANL2MB basis set, alongside Becke's three-parameter hybrid method and the Lee-Yang-Parr (B3LYP) functional.[13,14] The adsorption energy and the Highest Occupied Molecular Orbital-Lowest Unoccupied Molecular Orbital (HOMO-LUMO) gap for pure CuO, rGO, CuO/rGO composites, and CO2 were calculated using the Gaussian 16 software package and visualized with GaussView 6.1. Additionally, GaussSum Version 3.0 facilitated the calculation of molecular orbits' group contributions and the generation of Total Density of States (TDOS) spectra.[15] In this context, the ionization potential (IP) is defined as the energy required to remove one electron from the system at its lowest energy state. The difference in energy between the neutral and anionic forms is referred to as the electron affinity. The quantum molecular characteristics for all models were determined using the following equations::

$E_{gap}= E_{LUMO}- E_{HOMO}$ (1)

$\mu=(E_{LUMO}+ E_{HOMO})/2$ (2)

$\eta= (E_{LUMO}- E_{HOMO})/2$ (3)

$S= 1/2\eta$ (4)

where, 'μ' is chemical potential, 'χ' =-μ is electronegativity, 'η' is hardness, and 'S' is softness.

First principle calculations

The Density Functional Theory (DFT) study was conducted to theoretically correlate with the experimental findings by optimizing and determining the structural stability, adsorption behavior, and electrical characteristics of CO_2 on pure CuO, rGO, and CuO/rGO heterostructures. The investigation extended to various theoretical parameters before and during the interaction with CO_2, offering insights into the material's response to gas exposure.

According to the comparative analysis summarized in **Table S4**, the adsorption energy of the CuO/rGO p-p heterojunction significantly exceeds that of both pure CuO and rGO. This highlights the enhanced interaction between CO_2 molecules and the

CuO/rGO heterostructure, attributing to its higher adsorption capacity. Notably, upon CO_2 adsorption, there is an observable increase in the HOMO-LUMO (Highest Occupied Molecular Orbital-Lowest Unoccupied Molecular Orbital) gap, escalating from 0.35 eV to 0.88 eV. Such an increase in the HOMO-LUMO gap indicates a wider depletion region, consequently leading to an enhanced resistance against electron flow and thereby reducing the conductivity of the nanocomposite.

The study also acknowledges the significant influence of surface configurations on the material's surface states, which directly affect its physical and chemical properties. Invoking the Bloch theorem, the research underscores the importance of examining point group symmetry to understand the material's electronic structure comprehensively. To delve deeper into the electronic state and charge transfer dynamics during the adsorption process, Total Density of State (TDOS) spectra were generated for both isolated CuO/rGO and CuO/rGO in the presence of CO2, as illustrated in **Figure S12a** and **S12b**. These TDOS spectra, produced using the GaussSum program, revealed that the gap between occupied and virtual orbitals—those levels whose energies correspond closely with excitation energies widens post-CO_2 interaction.

Figure S12b presents the HOMO and LUMO energy levels of CuO/rGO atoms before and after CO_2 interaction, shedding light on the alteration of their state. The interaction with CO_2 gas notably increases the potential difference, which inherently diminishes the electron flow within the material. This interaction correlates with a decrease in the band gap and an increase in material resistance when exposed to CO_2, as depicted in **Figure S12**, demonstrating a clear link between the electronic structure changes induced by CO_2 adsorption and the observed increase in sensor resistance.

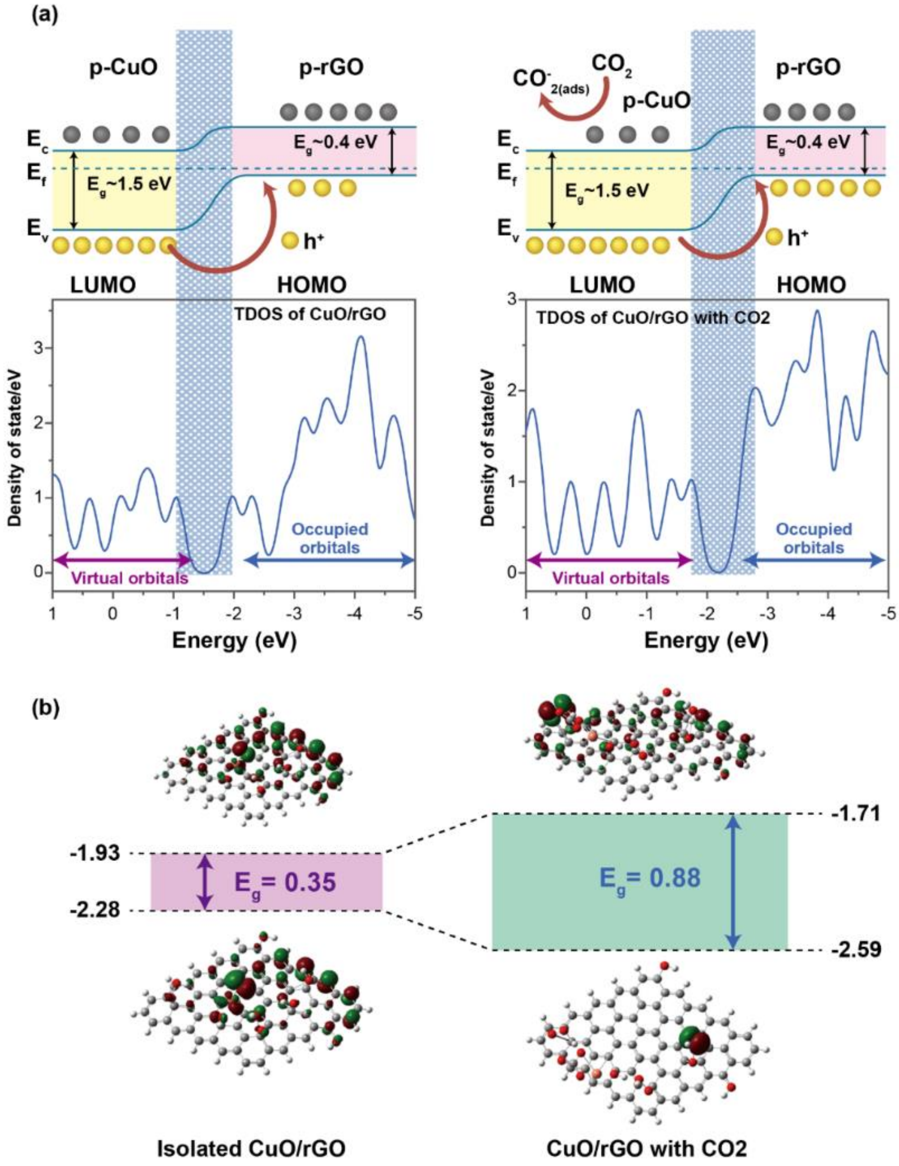

Figure S12. *(a) TDOS spectra with energy band diagram of CuO/rGO before and after interaction with CO_2 and (b) Variations in the HOMO-LUMO levels of CuO/rGO before and after interaction with CO_2.*

Table S4: *Theoretical parameters of CuO, rGO and CuO/rGO heterostructures before and after interaction with CO_2.*

Samples	CuO		rGO		CuO/rGO	
DFT Parameters	before interaction	after interaction with CO_2	before interaction	after interaction with CO_2	before interaction	after interaction with CO_2
Adsorption energy (eV)	-	-1.63	-	1.63	-	-4.08
Ionization potential (eV)	6.77	5.55	2.03	1.90	2.28	2.59
Electron affinity (eV)	2.73	2.54	1.66	1.42	1.93	1.71
HOMO–LUMO gap (E_{gap}) (eV)	4.04	3.01	0.37	0.48	0.35	0.88
Electronegativity (χ) (eV)	-4.75	-4.04	-1.84	-1.66	-2.10	-2.15
Hardness (η) (eV)	2.02	1.50	0.18	0.24	0.17	0.44
Softness (S) (eV)	0.24	0.33	2.7	2.08	2.85	1.13
Dipole moment (Debye)	0.22	1.81	4.46	5.89	13.42	13.60

References

(1) Najafi Nobar, S.Cu-BTC Synthesis, Characterization and Preparation for Adsorption Studies. Mater. Chem. Phys. 2018, 213, 343–351. https://doi.org/10.1016/j.matchemphys.2018.04.031.

(2) Yin, H.; Yu, X.-X.; Li, Q.-W.; Cao, M.-L.; Zhang, W.; Zhao, H.; Zhu, M.-Q.Hollow Porous CuO/C Composite Microcubes Derived from Metal-Organic Framework Templates for Highly Reversible Lithium-Ion Batteries. J. Alloys Compd. 2017, 706, 97–102. https://doi.org/10.1016/j.jallcom.2017.02.215.

(3) Yadav, A. A.; Lokhande, A. C.; Kim, J. H.; Lokhande, C. D.Enhanced Sensitivity and Selectivity of CO2 Gas Sensor Based on Modified La2O3 Nanorods. J. Alloys Compd. 2017, 723, 880–886. https://doi.org/10.1016/j.jallcom.2017.06.223.

(4) Bhowmick, T.; Ghosh, A.; Nag, S.; Majumder, S. B.Sensitive and Selective CO2 Gas Sensor Based on CuO/ZnO Bilayer Thin-Film Architecture. J. Alloys Compd. 2022, 903, 163871. https://doi.org/10.1016/j.jallcom.2022.163871.

(5) Zhang, C.; Liu, K.; Zheng, Z.; Debliquy, M.Defect Engineering of Nanostructured ZnSnO3 for Conductometric Room Temperature CO2 Sensors. Sensors

Actuators B Chem. 2023, 384, 133628. https://doi.org/10.1016/j.snb.2023.133628.

(6) Son, M.; Pak, Y.; Chee, S.-S.; Auxilia, F. M.; Kim, K.; Lee, B.-K.; Lee, S.; Kang, S. K.; Lee, C.; Lee, J. S.; Kim, K. K.; Jang, Y. H.; Lee, B. H.; Jung, G.-Y.; Ham, M.-H.Charge Transfer in Graphene/Polymer Interfaces for CO2 Detection. Nano Res. 2018, 11 (7), 3529–3536. https://doi.org/10.1007/s12274-017-1857-z.

(7) Nemade, K. R.; Waghuley, S. A.Role of Defects Concentration on Optical and Carbon Dioxide Gas Sensing Properties of Sb2O3/Graphene Composites. Opt. Mater. (Amst). 2014, 36 (3), 712–716. https://doi.org/10.1016/j.optmat.2013.11.024.

(8) Nemade, K. R.; Waghuley, S. A.Highly Responsive Carbon Dioxide Sensing by Graphene/Al2O3 Quantum Dots Composites at Low Operable Temperature. Indian J. Phys. 2014, 88 (6), 577–583. https://doi.org/10.1007/s12648-014-0454-1.

(9) Prim, A.; Pellicer, E.; Rossinyol, E.; Peiró, F.; Cornet, A.; Morante, J. R.A Novel Mesoporous CaO□Loaded In 2 O 3 Material for CO 2 Sensing. Adv. Funct. Mater. 2007, 17 (15), 2957–2963. https://doi.org/10.1002/adfm.200601072.

(10) Zhang, W.; Xie, C.; Zhang, G.; Zhang, J.; Zhang, S.; Zeng, D.Porous LaFeO3/SnO2 Nanocomposite Film for CO2 Detection with High Sensitivity. Mater. Chem. Phys. 2017, 186, 228–236. https://doi.org/10.1016/j.matchemphys.2016.10.048.

(11) Amarnath, M.; Gurunathan, K.Highly Selective CO2 Gas Sensor Using Stabilized NiO-In2O3 Nanospheres Coated Reduced Graphene Oxide Sensing Electrodes at Room Temperature. J. Alloys Compd. 2021, 857, 157584. https://doi.org/10.1016/j.jallcom.2020.157584.

(12) Singh, A.; Singh, S.; Yadav, B. C.Gigantic Enhancement in Response of Heterostructured CeO2/CdS Nanospheres Based Self-Powered CO2 Gas Sensor: A Comparative Study. Sensors Actuators B Chem. 2023, 377, 133085. https://doi.org/10.1016/j.snb.2022.133085.

(13) Miehlich, B.; Savin, A.; Stoll, H.; Preuss, H.Results Obtained with the Correlation Energy Density Functionals of Becke and Lee, Yang and Parr. Chem. Phys. Lett. 1989, 157 (3), 200–206. https://doi.org/10.1016/0009-2614(89)87234-3.

(14) Becke, A. D.Density-Functional Thermochemistry. III. The Role of Exact Exchange. J. Chem. Phys. 1993, 98 (7), 5648–5652. https://doi.org/10.1063/1.464913.

(15) O'boyle, N. M.; Tenderholt, A. L.; Langner, K. M.Cclib: A Library for Package□independent Computational Chemistry Algorithms. J. Comput. Chem. 2008, 29 (5), 839–845. https://doi.org/10.1002/jcc.20823.
